# Advances in the Pharmacological Management of Diabetic Nephropathy: A 2022 International Update

**DOI:** 10.3390/biomedicines11020291

**Published:** 2023-01-20

**Authors:** Rosaria Vincenza Giglio, Angelo Maria Patti, Ali Abbas Rizvi, Anca Panta Stoian, Marcello Ciaccio, Nikolaos Papanas, Andrej Janez, Alper Sonmez, Maciej Banach, Amirhossein Sahebkar, Manfredi Rizzo

**Affiliations:** 1 Department of Biomedicine, Neuroscience and Advanced Diagnostics, University of Palermo, 90127 Palermo, Italy; 2 Department of Laboratory Medicine, University Hospital, 90127 Palermo, Italy; 3 Internal Medicine Unit, “Vittorio Emanuele II” Hospital, Castelvetrano, 91022 Trapani, Italy; 4 Department of Medicine, University of Central Florida College of Medicine, Orlando, FL 32827, USA; 5 Faculty of Medicine, Diabetes, Nutrition and Metabolic Diseases, Carol Davila University, 050474 Bucharest, Romania; 6 Diabetes Centre, Second Department of Internal Medicine, Democritus University of Thrace, University Hospital of Alexandroupolis, 68132 Alexandroupoli, Greece; 7 Department of Endocrinology, Diabetes and Metabolic Diseases, University Medical Centre, University of Ljubljana, 1000 Ljubljana, Slovenia; 8 Department of Endocrinology and Metabolism, Gulhane Medical School, University of Health Sciences, Ankara 34668, Turkey; 9 Polish Mother’s Memorial Hospital Research Institute, Rzgowska 281/289, 93-338 Lodz, Poland; 10 Department of Preventive Cardiology and Lipidology, Medical University of Lodz, 91-347 Lodz, Poland; 11 Cardiovascular Research Centre, University of Zielona Gora, 65-046 Zielona Gora, Poland; 12 Applied Biomedical Research Center, Mashhad University of Medical Sciences, Mashhad 9177948954, Iran; 13 Biotechnology Research Center, Pharmaceutical Technology Institute, Mashhad University of Medical Sciences, Mashhad 9177948954, Iran; 14 Department of Health Promotion Sciences Maternal and Infantile Care, Internal Medicine and Medical Specialties (PROMISE), University of Palermo, 90133 Palermo, Italy

**Keywords:** diabetes mellitus, kidney disease, innovative therapy, cardiovascular risk, kidney protection

## Abstract

Diabetic nephropathy (DN) is the leading cause of end-stage renal disease (ESRD) worldwide. Its pathogenesis encompasses functional alterations involving elevated intraglomerular and systemic pressure, increased activity of the renin-angiotensin system (RAS) and oxidative stress, and the eventual development of renal fibrosis. The management of DN involves the optimization of blood pressure (BP) and blood glucose targets. However, treatment of these risk factors slows down but does not stop the progression of DN. Innovative pharmacologic therapies for dyslipidemia and type 2 diabetes mellitus (T2DM) could play a key role in bridging this gap and attenuating the residual risk of DN beyond traditional risk factor management. Glucagon-like peptide-1 receptor agonists (GLP-1 RAs), sodium-glucose cotransporter-2 inhibitors (SGLT-2is), and inhibitors of mineralocorticoid receptor-mediated sodium reabsorption are recently introduced drug classes that have been shown to have positive effects on kidney function in individuals with T2DM. The aim of this review is to provide an update on the therapeutic options available in order to prevent or slow the onset and progression of DN in diabetic patients.

## 1. Introduction

Diabetic kidney disease (CKD), or diabetic nephropathy (DN), is a major complication that takes a significant toll on communities globally [1]. In general, the natural history of DN consists of three stages: normoalbuminuria, microalbuminuria, and macroalbuminuria. Glomerular hyperfiltration is considered a hallmark of early disease. The appearance of albuminuria is associated with a progressive decline in the glomerular filtration rate (GFR) that parallels the degree of albumin excretion in addition to BP and glucose; a reduction in GFR may be due to other factors such as female gender, obesity, and the presence of hypertriglyceridemia, thus explaining the fact that the heterogeneity of the DN phenotype is not closely linked to its typical histological lesions [2].

The pathogenesis of DN encompasses a number of functional alterations involving increased intraglomerular pressure, stimulation of the renin-angiotensin system (RAS), oxidative stress, and fibrotic changes that are superimposed on an innate genetic predisposition [3]. In addition, increased circulating inflammatory mediators (adhesion molecules and chemokines) and intrarenal immune cell infiltration are observed in patients with DN [4,5]. This review focuses on innovative pharmacologic treatments that promise to provide breakthroughs in the management of diabetic nephropathy.

Treatment strategies for the management of DN include (a) intensive blood glucose control; (b) attainment of blood pressure goals with the inclusion of angiotensin II receptor blockers (ARBs) or angiotensin-converting enzyme (ACE)-inhibitors in the pharmacologic regimen; (c) weight management with dietary caloric restriction and aerobic physical exercise; (d) dietary protein restriction; (e) smoking cessation; (f) lipid management with statin therapy as the preferred choice; and (g) avoidance of nephrotoxic drugs such as contrast agents, antibiotics, and nonsteroidal anti-inflammatory drugs (NSAIDs) [6,7]. One of the significant breakthroughs in the field of DN treatment has been the introduction of finerenone [8], a nonsteroidal selective mineralocorticoid receptor antagonist (MRA). This drug has pharmacokinetic and clinical effects that are different with respect to steroidal MRAs, such as eplerenone and spironolactone [9,10,11,12].

Novel pharmacologic approaches are on the horizon to slow the progress and evolution of DN and thereby protect the kidneys [13,14]. SGLT-2is are drugs capable of lowering blood sugar by increasing the urine excretion of glucose. However, they have also demonstrated positive effects regarding the cardiovascular and renal systems in T2DM subjects and in those with renal or cardiological disease without T2DM. These drugs can reduce the risk of dialysis and mortality from kidney diseases as they are able to block CKD progression.

## 2. Pathogenesis and Course of DN

DN begins with glomerular hyperfiltration, which abates with the onset of early renal damage [1,3,6,7]. This is accompanied by mild systemic hypertension, which worsens over time [1,3,6,7]. Increased albumin excretion in the urine from early renal damage is manifested by microalbuminuria (30–299 mg/24 h) followed by macroalbuminuria (≥300 mg/24 h). Patients go from an asymptomatic stage to fluid retention, which is usually accompanied by clinically overt edema. In advanced stages, patients may exhibit the symptoms of uremia [1,3,7].

Several factors contribute to the pathophysiology of DN. Hyperglycemia leads to the increased formation of advanced glycation end products and a proinflammatory state, which have been correlated with a progressive reduction in GFR [1,3,6,7]. The former becomes manifest with increased adhesion molecules, infiltrating cells, reactive oxygen species (ROS), cytokines, and metalloproteinases [15,16]. Systemic hypertension can compromise the glomerular and functional structure due to a disequilibrium in the efferent and afferent arteriolar pressure [15,16,17]. Dyslipidemia, obesity, and insulin resistance also aggravate renal hemodynamics [17].

## 3. Search Strategy

**Figure 1:**
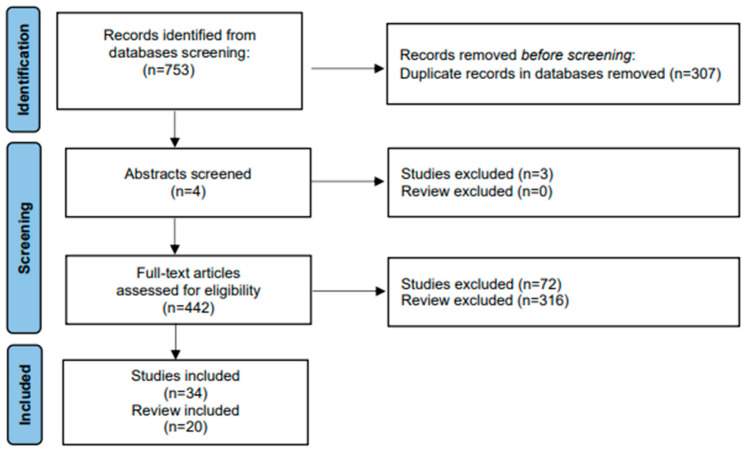
Flow chart related to the selection methodology of the studies included in the review.

We searched electronic databases (MEDLINE (1975–2022), EMBASE and SCOPUS (2000–2022)) and Web of Science Core Collection (1997-present) using combinations of the following keywords: diabetes, diabetic nephropathy, incretins, dipeptidyl peptidase-4 inhibitors, glucagon-like peptide-1 receptor agonists, sodium-glucose transporter-2 inhibitors, kidney disease, therapy, treatment. The methodology used in extracting the relevant studies and reviews is summarized in the flow chart (Figure 1).

## 4. Pharmacologic Management of DN: Current and Emerging Evidence

### 4.1. Conventional Therapy in Diabetic Kidney Disease

Blood pressure (BP) control by renin-angiotensin system (RAS) blockade reduces proteinuria and effectively slows down the evolution of diabetic and nondiabetic nephropathies through local and/or systemic actions [7]. The optimization of glycemic control with antihyperglycemic medications, including insulin therapy, is fundamentally important. However, care is needed to adjust the doses or avoid certain drugs due to declining renal function [18,19]. Statins, especially simvastatin, cerivastatin, and rosuvastatin, can be used in diabetic nephropathy, as they reduce albuminuria, urinary endothelin, and even blood pressure [20]. Unfortunately, conventional multifactorial therapy addressing blood pressure, glycemic, and lipid control is challenging to maintain and not efficacious enough to reduce the incidence of DN. Statins might exert significant renoprotective effects that depend partly on the duration of therapy [21].

Positive results in terms of primary prevention were noted in the Action in Diabetes and Vascular Disease: Preterax and Diamicron Modified Release Controlled Evaluation (ADVANCE; ClinicalTrials.gov Identifier: NCT00949286) study [22] in the Bergamo Nephrologic Diabetic Complications (BENEDICT; ClinicalTrials.gov Identifier: NCT00235014) trial [23] and the Randomized Olmesartan and Diabetes Microalbuminuria Prevention ROADMAP study (ClinicalTrials.gov Identifier: NCT00185159) [24]. In the BENEDICT study, treatment with ACE-is (trandolapril or trandolapril and verapamil in combination) reduced the cumulative incidence of microalbuminuria over the 3.6-year follow-up. In the ADVANCE Blood Pressure trial, patients on active treatment with perindopril/indapamide had significantly lower blood pressure values with lower microalbuminuria values [22]. In the ROADMAP study, treatment with olmesartan delayed the onset of microalbuminuria [24]. In the Renin-Angiotensin System Study (RASS), enalapril and losartan decreased diabetic retinopathy progression. In patients with microalbuminuria, therapy aims to prevent progression to clinical albuminuria and promote regression toward normoalbuminuria (secondary prevention). Therefore, patients with microalbuminuria need to be treated with ACE-is or ARBs regardless of blood pressure. In microalbuminuric patients with T2DM, both angiotensin receptor antagonists (IRMA 2—Irbesartan Microalbuminuria Type 2 Diabetes Mellitus in Hypertensive Patients [25]; MARVAL—Micro Albuminuria Reduction with VALsartan Study Investigators [26]) and ACE inhibitors (ADVANCE) [22] decrease the risk of developing proteinuria. In addition, in subjects with T2DM and microalbuminuria, both RAS blockers increased the likelihood of regression to normoalbuminuria.

### 4.2. Advances in Treatment of Diabetic Kidney Disease

In recent years, the development of new hypoglycemic drugs has dramatically enriched the therapeutic options available for treating T2DM. Notably, some of these new agents, such as SGLT2is and GLP-1Ras, not only mitigate hyperglycemia but also reduce cardiovascular complications and protect kidney function [27,28,29,30]. Although insulin is the only medication available for subjects with T1DM, some of the innovative antidiabetic drugs used approved for T2DM have also shown some beneficial effects in T1DM through their pleiotropic actions [31,32].

#### 4.2.1. Glucagon-Like Peptide-1 Receptor Agonists

Glucagon-like peptide-1 receptor agonists (GLP-1RAs) improve kidney outcomes in T2DM. They improve renal tubular function and increase natriuresis and diuresis [33]. The effect of GLP-1RAs on renal hemodynamics is ascribed to the improved equilibrium between afferent vasodilation and efferent vasoconstriction [33]. The tubular transporter most likely mediating the natriuretic effects of GLP-1 is the sodium–hydrogen antiporter, also known as sodium hydrogen exchanger 3 (NHE3), which is found on the edge of the brush of the proximal tubule. It is tied to a formulation that also includes DPP-4 [34]. NHE3 is inhibited by decreased postprandial levels of glucagon [34], insulin [35], or glucose, which leads to downregulation and inhibition of NHE3 and SGLT-1/SGLT-2 [17,18]. The renal protection mechanism is postulated to be linked to the diuretic and natriuretic effect of GLP-1. GLP-1RAs under physiologic conditions seem to induce glomerular hyperfiltration with an increase in GFR. A direct effect of inducing vasodilation of the afferent arteriole is mediated by GLP-1RAs of the glomerular vascular smooth muscle cells. Interestingly, beyond the endocrine pancreas, GLP-1 receptor mRNA has been localized in the central and peripheral nervous systems, gastrointestinal tract, cardiovascular system, kidneys, and lungs [36]. Therefore, it is plausible to assume that, in addition to their glucose-lowering actions, these drugs have anti-inflammatory and antioxidant effects which could directly or indirectly improve kidney function.

The Evaluation of Lixisenatide in Acute Coronary Syndrome (ELIXA; ClinicalTrials.gov Identifier: NCT01147250) study studied patients with T2DM and a recent acute coronary event (unstable angina or myocardial infarction). Twenty-five percent of subjects included in the group treated with lixisenatide and twenty-two percent in the placebo group had a reduction in eGFR (<6.0 mL/min/1.73 m^2^) [37]. No statistically significant differences were observed in eGFR decline with lixisenatide treatment compared to placebo in general and after adjustment for baseline albuminuria status. However, a decrease in the incidence of macroalbuminuria onset was seen (HR 0.77; 95% CI: 0.62, 0.96; *p* = 0.0174), as well as a lower urinary albumin-to-creatinine ratio (UACR) in lixisenatide-treated patients with micro- (−21.10%, −42.25 to 0.04; *p* = 0.0502) or macroalbuminuria (−39.18%, −68.53 to −9.84, *p* = 0.0070) [37].

In the Liraglutide Effect and Action in Diabetes: Evaluation of Cardiovascular Outcome Results-A Long Term Evaluation (LEADER; ClinicalTrials.gov Identifier: NCT01179048) trial with liraglutide 1.8 mg, the renal outcomes studied included new-onset persistent macroalbuminuria, persistent doubling of serum creatinine, kidney failure, and death due to renal disease. A reduction in adverse renal outcomes was observed (HR 0.78, 95% CI: 0.67–0.92). There was a reduction in new-onset macroalbuminuria (HR 0.74, 95% CI 0.60–0.91, *p* = 0.004) and a reduction in the risk of major adverse CV events and all-cause mortality in comparison to placebo in patients with DCKD (eGFR < 60 mL/min/1.73 m^2^ and UACR > 30 mg/g) [38,39]. In addition, subcutaneous treatment with semaglutide demonstrated a decrease in the incidence of new cases of nephropathy or its aggravation in the Semaglutide and Cardiovascular Outcomes in Patients with Type 2 Diabetes Cardiovascular Outcome (SUSTAIN-6; ClinicalTrials.gov Identifier: NCT01720446) study (HR 0.64 (95% CI 0.46–0.88, *p* = 0.05)). This effect was due to a decrease in new-onset macroalbuminuria (0.973 with semaglutide 0.5 mg, 0.858 with semaglutide 1.0 mg). Posthoc analysis of SUSTAIN 1–5 and 7 demonstrated a 30% decrease in albuminuria and al regression to micro- or normoalbuminuria in all grades of albuminuria [40].

In the 2017 Exenatide Study of Cardiovascular Event Lowering (EXSCEL; ClinicalTrials.gov Identifier: NCT01144338) trial evaluating the cardiovascular safety of exenatide, in the composite primary outcome of the first occurrence of cardiovascular death, nonfatal myocardial infarction, or nonfatal stroke, no significant difference in the decline of the eGFR, Renal Replacement Therapy (RRT) or renal death was observed. The occurrence of macroalbuminuria was lower in the exenatide group (2.2%) than in the placebo (2.5%) (HR 0.87 (95% CI: 0.70–1.07)) [41]. In the Cardiovascular Safety of Oral Semaglutide in Subjects with Type 2 Diabetes (PIONEER-6; ClinicalTrials.gov Identifier: NCT02692716) trial, the main objective was evaluating the cardiovascular safety of oral semaglutide; 26.9% of the study’s patients had an eGFR < 60 mL/min/1.73 m^2^. No statistically significant differences were found in eGFR decline and renal death; in PIONEER-5, safety was demonstrated for subjects with a moderate eGFR reduction (30–59 mL/min/1.73 m^2^) [42,43].

Renal outcomes were also evaluated in A Study Comparing Dulaglutide with Insulin Glargine on Glycemic Control in Participants with Type 2 Diabetes and Moderate or Severe Chronic Kidney Disease (AWARD-7; ClinicalTrials.gov Identifier: NCT01621178). Patients with moderate-to-severe T2DM and CKD were treated with insulin and an angiotensin receptor blocker or angiotensin-converting enzyme inhibitor at the maximum dose along with dulaglutide injected once weekly. eGFR was greater with dulaglutide 1.5 mg (34.0 mL/min/1.73 m^2^; *p* = 0.005 compared to insulin glargine) and dulaglutide 0.75 mg (3.3.8 mL/min/1.73 m^2^; *p* = 0.009 versus insulin glargine) versus insulin glargine (31.3 mL/min/1.73 m^2^) after 52 weeks of therapy [44].

Patients with microalbuminuria showed a more evident link between dulaglutide therapy and a reduced decline in eGFR [44].

In the Researching Cardiovascular Events with a Weekly Incretin in Diabetes (REWIND; ClinicalTrials.gov Identifier: NCT01394952) study, the renal outcomes studied consisted of new-onset macroalbuminuria, sustained eGFR decline of 30% or more, and a need for RRT. A composite of these endpoints occurred in 17.1% of dulaglutide (1.5 mg) patients and 19.6% in the not-treated group (HR 0.85, 95% CI 0.77–0.93; *p* = 0.0004) [45]. The Research Study to See How Semaglutide Works Compared to Placebo in People with Type 2 Diabetes and Chronic Kidney Disease (FLO.W; ClinicalTrials.gov Identifier: NCT03819153) trial is an ongoing study evaluating the effect of semaglutide in patients with T2DM and CKD. It is the first study to have a renal outcome as the main endpoint which includes eGFR decline ≥ 50% from baseline, reaching kidney failure, or renal death from CV or kidney disease. The study is expected to conclude in August 2024 [46].

#### 4.2.2. Sodium–Glucose Cotransporter 2 Inhibitors

SGLT-2 inhibitors are drugs that act on transporters located in the proximal renal tubule, thereby reducing the reabsorption of glucose [47]. Patients with reduced eGFR have an attenuated hypoglycemic response because the reabsorption of glucose at the level of the proximal tubule is linearly associated with the glucose level in the blood and the quantity that the glomerulus can filter is therefore limited [48]. The loss of energy substrates with glycosuria produces weight loss, while water loss with diuresis leads to volume depletion and blood pressure reduction [47]. Analyses of the secondary outcomes in clinical studies have revealed strong renal protective effects and a retardation in the progression of renal pathology [49,50].

Several cardiovascular outcome studies in subjects with T2DM have shown a decreased risk of progression of CKD with SGLT2 inhibitors empagliflozin (in EMPA REG OUTCOME; ClinicalTrials.gov Identifier: NCT01131676) [49], canagliflozin (in CANVAS; ClinicalTrials.gov Identifier: NCT01032629) [51], and dapagliflozin (in DECLARE –TIMI 58; ClinicalTrials.gov Identifier: NCT01730534) [50]. In addition, a notable decrease in the risk of CKD progression was identified with canagliflozin in CKD subjects (CANVAS program; ClinicalTrials.gov Identifier: NCT01032629) [51] and with dapagliflozin in subjects with CKD or nondiabetic CKD (in DAPA-CKD; ClinicalTrials.gov Identifier: NCT03036150 and DECLARE-TIMI 58;) [52,53,54]. Furthermore, in the CANVAS program of randomized clinical trials, patients with albuminuric T2DM and CKD (eGFR 30 to <9.0 mL/min/1.73 m^2^; UACR ≥ 30.0 to 500.0 mg/g) who received canagliflozin at a dose of 100 mg once a day, a statistically significant reduction of 30% in the primary endpoint (ESKD, doubling the serum creatinine level, renal or cardiovascular cause of death) was observed compared to the untreated group (*p* < 0.001) [51]; at the same time, there was a statistically significant reduction of 32% in the risk of ESKD (*p* = 0.002) and a 34% reduction in the doubling of creatinine and renal death (*p* < 0.001) [51].

The DAP.A-CKD trial demonstrated a reduction of 39% in the primary endpoint (eGFR, ESKD, or renal or cardiovascular death) (*p* < 0.001) compared to the untreated group in subjects who had a chronic renal failure with or without T2DM (67.5% and 32.5%, respectively) [53]. DAPA-CKD was the first study to show a reduced risk of all-cause mortality in subjects with chronic renal failure but improved renal pathology outcomes with an SGLT2i agent [55].

In the DECLARE-TIMI trial of 58 subjects with CKD and T2DM receiving dapagliflozin compared to subjects with CKD but without diabetes, a reduction in cardiorenal outcomes (at least 40% sustained decline in eGFR > 60 mL/min/1.73 m^2^, ESKD or death from renal or cardiovascular causes) was noted [52,53,54]: the group treated with dapagliflozin showed a significantly decreased frequency of cardiorenal outcomes [HR 0.76 (95% CI 0.67–0.87); *p* < 0.0001] and kidney-specific outcomes [HR 0.53 (95% CI 0.43–0.66); *p*< 0.0001] with respect to the untreated group; the dapagliflozin group also had a significantly reduced frequency of composite cardiorenal outcome [HR 0.76 (95% CI 0.67–0.87); *p* < 0.0001] and kidney-specific outcomes [HR 0.53 (95% CI 0.43–0.66); *p* < 0.0001] compared to the placebo group [53]. Statistically significant decreases were observed both in cardiorenal outcome among subgroups with UACR ≥ 30 mg/g (*p* < 0.0125) but also in renal outcome in all UACR groups (*p* < 0.05) [54].

In the ESC guidelines of 2019 [56], the study Canagliflozin and Renal Events in Diabetes with Established Nephropathy Clinical Evaluation (CREDENCE; ClinicalTrials.gov Identifier: NCT02065791) is cited [57]. This study evaluated diabetic subjects with eGFR 3.0 to <9.0 mL/min/1.73 m^2^ treated with canagliflozin 100 mg/day or for the untreated group; the safety committee terminated the study prematurely after an analysis demonstrated superiority. The primary outcome that including a doubling of serum creatinine level, composite of end-stage kidney pathology, or renal or CV death was decreased by 30%, and secondary outcomes showed positive benefits with the use of canagliflozin. These results in a high-risk population of diabetic with renal insufficiency subjects confirm the observations of secondary outcomes in CVOTs and prove the necessity to use SGLT2is in the management of T2DM, CKD, and CVD. In addition, some of the GLP-1RAs may offer modest kidney beneficium [58]: a meta-analysis evaluating studies conducted with liraglutide, semaglutide, exenatide, and lixisenatide showed a decrease in the macroalbuminuria progress and the risk of contrary renal consequences due to decreased proteinuria [59]. Based on the results of the CREDENCE, DECLARE–TIMI 5.8, and DAPA-CKD studies, canagliflozin would benefit subjects with diabetic nephropathy and T2DM, with albuminuria > 300 mg/day and an eGFR ≥ 30 mL/min/1.73 m^2^, and dapagliflozin would be helpful in chronic renal failure patients with an eGFR ≥ 25 mL/min/1.73 m^2^ at risk of CKD progression. Though canagliflozin decreased the risk of the evolution of chronic renal failure, cardiovascular death, and hospitalization for heart failure (HHF) only in diabetic subjects with albuminuria ≥ 300 mg/g, dapagliflozin is designed to decrease the risk of evolution of CKD and HHF regardless of albuminuria in all CKD subjects with or without T2DM, as well as decrease the risk of cardiovascular and HHF death in heart failure subjects with a low ejection fraction [55].

SGLT2is reduce in glomerular hyperfiltration, possibly due to an increase in preglomerular vasoconstriction and a reduction in postglomerular vascular resistance [60]; this eGFR reduction is similar to that induced by adenosine [61] through preglomerular vasoconstriction and postglomerular vasodilation [60]. The CREDENCE and DAPA-CKD studies demonstrated that canagliflozin or dapagliflozin resulted in a mean eGFR reduction of 3.7 mL/min/1.73 m^2^ within three weeks of therapy and 4.0 mL/min/1.73 m^2^ within two weeks. This reduction persisted and remained constant throughout the year and was lower with SGLT-2is drugs than in the untreated control group [53,62]. A decrease in eGFR of more than 10% was observed in subjects with more advanced stages of chronic renal failure with reduced diuresis being treated with diuretics, but this did not influence renal or cardiovascular outcomes [63].

#### 4.2.3. Dipeptidyl Peptidase-4 Inhibitors

DPP-4is boost the levels of GLP-1 and GIP, which inhibit the release of glucagon, increasing insulin secretion, reducing gastric emptying, and decreasing blood glucose levels. They are suitable for subjects with advanced T2DM and kidney dysfunction [64]. In addition, DPP-4 is widely expressed in epithelial and endothelial tissue of the proximal renal tubules, and it interacts with the components of the extracellular matrix (fibronectin and collagen) [65]. DPP-4is attenuate renal fibrosis and podocyte damage and reduce proteinuria levels independently of glycemic control [66].

In the Saxagliptin Assessment of Vascular Outcomes Recorded in Patients with Diabetes Mellitus Thrombolysis in Myocardial Infarction (SAVOR TIMI 53; ClinicalTrials.gov Identifier: NCT01107886) study [64], saxagliptin was associated with a reduction in albuminuria level irrespective of glucose control; there was no statistically significant difference during follow-up in eGFR or the prevalence of renal endpoints [64]. In the Trial Evaluating Cardiovascular Outcomes with Sitagliptin (TECOS; ClinicalTrials.gov Identifier: NCT00790205) study on sitagliptin, significant reductions in sitagliptin-associated albuminuria were observed without no changes in eGFR [67].

The Cardiovascular and Renal Microvascular Outcome Study with Linagliptin (CARMELINA; ClinicalTrials.gov Identifier: NCT01897532) showed cardiovascular safety of the drug with no significant differences in renal outcomes except for less progression of albuminuria than for the placebo [68]. The Efficacy, Safety, and Modification of Albuminuria in Type 2 Diabetes Subjects with Renal Disease with LINAgliptin (MARLINA-T2D; ClinicalTrials.gov Identifier: NCT01792518) trial followed patients with elevated urine albumin excretion (albumin-to-creatinine ratio greater than 30 mg/g) who were treated with linagliptin. This drug improved glycemic control without showing a statistically significant effect on albuminuria values [69]. The impact of DPP-4 is on cardiovascular outcomes in patients with diabetic nephropathy has been neutral though the agents were found to be safe from the renal standpoint [68].

#### 4.2.4. Finerenone: A New Mineralocorticoid Receptor Antagonist

The Finerenone in Reducing Kidney Failure and Disease Progression in Diabetic Kidney Disease (FIDELIO-DKD; ClinicalTrials.gov Identifier: NCT02540993) and the Finerenone in Reducing Cardiovascular Mortality and Morbidity in Diabetic Kidney Disease (FIGARO-DKD; ClinicalTrials.gov Identifier: NCT02545049) trials were two randomized controlled, phase three trials that investigated the beneficial effects of finerenone on the renal and cardiovascular systems in patients with type 2 diabetes mellitus (T2DM) and CKD. FIDELIO-DKD and FIGARO-DKD had a primary and secondary endpoint but were inverted (renal and cardiovascular endpoint, cardiovascular and renal endpoint, respectively, but with less pronounced CKD in the latter trial) [10,11]; the use of finerenone alone and in association with glucagon-like peptide-1 receptor agonists (GLP-1Ras) or sodium–glucose cotransporter two inhibitors (SGLT2is) has been shown to have cardiovascular benefits [12]. In addition, the FIDELITY meta-analysis of the FIGARO-DKD and FIDELIO-DKD trials found that finerenone resulted in a reduction in renal risk equal to or greater than 57% reduction in glomerular filtration rate (eGFR) and a reduction in the risk of irreversible kidney failure or damage; it also demonstrated a reduction in cardiovascular risk by evaluating time to non-fatal myocardial infarction, non-fatal stroke and hospitalization for heart failure and cardiovascular death [12].

#### 4.2.5. Novel Lipid-Lowering Drugs

CKD patients have high concentrations of LDL-C, very low-density lipoprotein (VLDL), and lipoprotein (a), as well as low concentrations of Apo A-I [70]. According to the recent guidelines for the management of dyslipidemia, CKD is one of the conditions correlated with a high risk of atherosclerotic cardiovascular disease (ASCVD); the LDL-C target is below 55 for secondary prevention or 70 mg/dL for primary prevention [71].

The management of dyslipidemia in CKD patients remains challenging. Patients with less severe renal insufficiency appear to have a consistent benefit from lipid-lowering treatment with statins in both primary and secondary prevention [72]; however, despite the positive effect of lipid-lowering drugs, decreased renal function can lead to statin-related side effects, including myopathy. In advanced CKD subjects, especially in subjects with end-stage renal disease (ESRD) undergoing hemodialysis, statins have not shown the ability to reduce cardiovascular events [73].

The use of combination lipid-lowering therapies to attain lower LDL cholesterol targets is an effective therapeutic approach in reducing the cardiovascular risk in subjects with CKD. Proprotein convertase subtilisin/kexin type 9 (PCSK9) is a fundamental modulator of LDL receptors in the liver [74,75]. PCKK-9 inhibitors exploit monoclonal antibodies or RNA interference to target the PCSK9 protein [76]. Clustered regularly interspaced short palindromic repeats (CRISPR) gene editing targeted at PCSK9 could be a promising tool for achieving the ambitious goal of a lifelong “fire and forget” mode to decrease LDL-C [77]. However, the use of multiple gene editing treatments and other biologics is still being explored. A maladaptive epigenetic response is an element of cardiovascular risk, and both chronic renal failure and T2DM augment it. Apabetalone, a selective modulator of the bromodomain and the transcription system of the extraterminal domain, reduces the incidence of major cardiovascular outcomes in renal patients [78].

PCSK9 has pleiotropic functions in the body, including trafficking of the epithelial sodium channel [75]. The Further Cardiovascular Outcomes Research with PCSK9 Inhibition in Subjects with Elevated Risk (FOURIER; ClinicalTrials.gov Identifier: NCT01764633) study investigated the potential benefit of treatment with PCSK9 inhibitors to decrease residual risk among subjects with ASCVD and CKD. The use of evolocumab was shown to be safe and effective overall eGFR ranges above 20 mL/min/1.73 m^2^; it is not known whether the benefits of evalocumab extend to patients at lower risk [79]. Data from clinical studies with Inclisiran in the ORION program (ClinicalTrials.gov Identifier: NCT03399370) showed the safety and efficacy of inclisiran in subjects with dyslipidaemia [76]. There were no substantial changes in renal function in subjects with CKD, while adverse outcomes were comparable in the Inclisiran and untreated groups [80].

## 5. Summary

**Figure 2:**
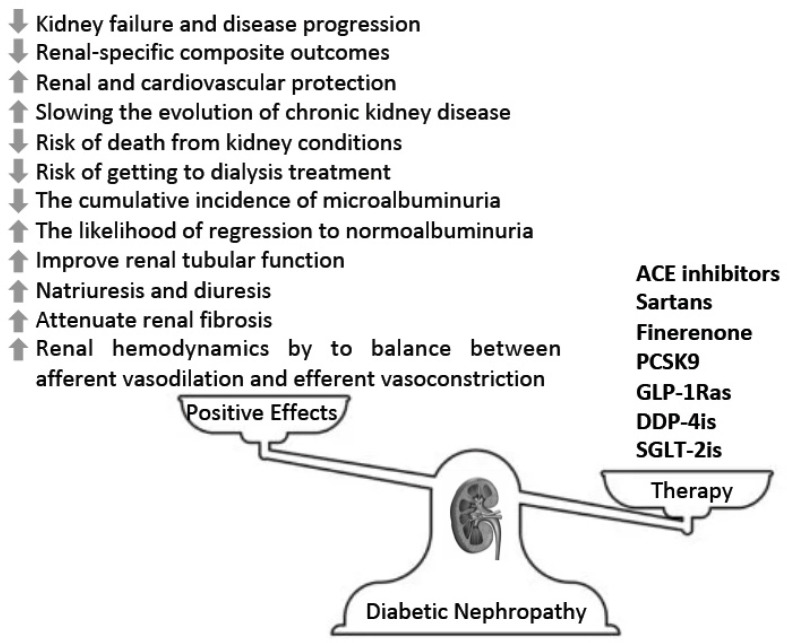
Main effects of traditional and innovative therapies on diabetic nephropathy. ACE, angiotensin converting enzyme; DPP-4is, dipeptidyl peptidase 4 inhibitors; GLP-1 Ras, glucagon-like peptide-1 receptor agonists; PCSK9, proprotein convertase subtilisin/kexin type 9; SGLT-2is, sodium glucose cotransporter 2 inhibitors.

The impact of traditional and emerging therapies on diabetic nephropathy is summarized in Figure 2. Appropriate management of DN, as with other vascular complications of diabetes, requires a multifactorial intervention consisting of lifestyle modification and the attainment of glucose, blood pressure, and lipid goals [81,82]. The role of pharmacologic agents that are useful in modulating these risk factors and are concurrently beneficial for DN is an important element of therapy. In this respect, the pleiotropic cardiovascular and renal actions of SGLT-2is and GLP-1RAs have come into focus, while specific renoprotective actions have been observed with the mineralocorticoid agent finerenone [82,83]. The 2022 American Diabetes Association/European Association for the Study of Diabetes (ADA/EASD) consensus statement [84] has emphasized the unique benefits of the former beyond their glucose-lowering effects.

The consensus report advises a patient-focused approach to selecting the suitable drug treatments for hyperglycemia [84]: For patients with chronic renal failure, the usage of an SGLT2 or GLP-1 RA inhibitor with confirmed CVD advantage is recommended regardless of the glycated hemoglobin level. In studies of cardiovascular outcomes, canagliflozin, dapagliflozin, empagliflozin, liraglutide, semaglutide, and dulaglutide all had a positive effect on CKD parameters. Dedicated renal outcome studies also proved the advantage of specific SGLT2is in reducing the incidence and progression of albuminuria in addition to the cardiovascular benefits [84]. We also need to highlight that some collateral effects are present; indeed, the side effect profile of SGLT-2is is characterized by urinary and genital infections, while that of GLP-1RAs includes gastrointestinal symptoms and a rare association with acute pancreatitis. Yet, these adverse effects are usually mild, transient, easily managed, and are outweighed by the cardiorenal benefits. In conclusion, the quest for the preservation of kidney function in diabetes is undergoing an exciting period, with a blend of traditional and new interventions holding promise in reducing the detrimental impact on our patients.

## 6. Conclusions

Nephropathy is considered a microvascular complication very frequently associated with diabetes; therefore, traditional therapies are not able to fully protect against the evolution of renal failure. Innovative treatments could be useful in preventing and treating diabetic nephropathy by modulating the underlying inflammatory process: A new field of research may be a challenge for SGLT-2 inhibitors or GLP-1 agonists and so forth. The main effects of traditional and innovative therapies on diabetic nephropathy are summarized in Figure 2. Further clinical trials are useful to study the possible reno-protective efficacy of new hypoglycemic drugs beyond RAS blockade.

## Data Availability

Not applicable.

## Abbreviations

ACE	Angiotensin Converting Enzyme
ADA	American Diabetes Association
ADVANCE	Action in Diabetes and Vascular Disease: Preterax and Diamicron Modified Release Controlled Evaluation
ARBs	Angiotensin II Receptor Blockers
AWARD-7	A Study Comparing Dulaglutide with Insulin Glargine on Glycemic Control in Participants with Type 2 Diabetes and Moderate or Severe Chronic Kidney Disease
ASCVD	Atherosclerotic Cardiovascular Disease
BENEDICT	Bergamo Nephrologic Diabetic Complications
BP	Blood Pressure
CANVAS	Canagliflozin Cardiovascular Assessment
CKD	Chronic Kidney Disease
CI	Confidence Interval
CREDENCE	Evaluation of the Effects of Canagliflozin on Renal and Cardiovascular Outcomes in participants with Diabetic Nephropathy
CRISPR	Clustered Regularly Interspaced Short Palindromic Repeats
DAPA-CKD	Dapagliflozin on Renal Outcomes and Cardiovascular Mortality in Patients with Chronic Kidney Disease
DECLARE-TIMI	Dapagliflozin and Cardiovascular Outcomes in Type 2 Diabetes
DN	Diabetic Nephropathy
DPP-4	Dipeptidyl Peptidase 4
DPP-4is	Dipeptidyl Peptidase 4 inhibitors
EASD	European Association for the Study of Diabetes
eGFR	estimated Glomerular Filtration Rate
ELIXA	Evaluation of Lixisenatide in Acute Coronary Syndrome
EMPA-KIDNEY	The Study of Heart and Kidney Protection with Empagliflozin, study of heart protection and kidneys with Empagliflozin
EMPA-REG	Empagliflozin cardiovascular outcome events in T2DM patients
ESRD	End-Stage Renal Disease
EXSCEL	Exenatide Study of Cardiovascular Event Lowering
FIDELIO-DKD	Finerenone in Reducing Kidney Failure and Disease Progression in Diabetic Kidney Disease
FIGARO-DKD	Finerenone in Reducing Cardiovascular Mortality and Morbidity in Diabetic kidney Disease
FLOWA	Research Study to See How Semaglutide Works Compared to Placebo in People with Type 2 Diabetes and Chronic Kidney Disease
GFR	Glomerular Filtration Rate
GLP-1	Glucagon-Like Peptide-1
GLP-1 Ras	Glucagon-Like Peptide-1 Receptor Agonists
HbA1c	Hemoglobin A1c
HR	Hazard Ratio
KIDNEY	The Study of Heart and Kidney Protection with Empagliflozin
ICAM1	Intercellular Adhesion Molecule 1
LDL-C	Low-Density Lipoproteins Cholesterol
LEADER	Liraglutide Effect and Action in Diabetes: Evaluation of Cardiovascular Outcome Results-A Long Term Evaluation
MACE	Major Adverse Cardiovascular Events
MRA	Mineralocorticoid Receptor Antagonist
NHE3	Sodium Hydrogen Exchanger 3
NSAIDs	Nonsteroidal Anti-Inflammatory Drugs
PCSK9	ProproteinConvertase Subtilisin/Kexin type 9
PIONEER-6	Cardiovascular Safety of Oral Semaglutide in Subjects with Type 2 Diabetes
RAS	Renin-Angiotensin System
RASS	Renin-Angiotensin System Study
RCTs	Randomized Controlled Trials
REWIND	Researching Cardiovascular Events with a Weekly Incretin in Diabetes
ROADMAP	Randomized Olmesartan and Diabetes Microalbuminuria Prevention
ROS	Reactive Oxygen Species
RRT	Renal Replacement Therapy
SGLT-2is	Sodium Glucose Cotransporter 2 inhibitors
SUSTAIN-6	Semaglutide and Cardiovascular Outcomes in Patients with Type 2 Diabetes Cardiovascular Outcome
T2DM	Type 2 Diabetes Mellitus
UACR	Urinary Albumin-To-Creatinine Ratio
VCAM1	Vascular Cell Adhesion Molecule 1
VLDL	Very Low-Density Lipoprotein
